# Bioinspired genotype–phenotype linkages: mimicking cellular compartmentalization for the engineering of functional proteins

**DOI:** 10.1098/rsfs.2015.0035

**Published:** 2015-08-06

**Authors:** Liisa D. van Vliet, Pierre-Yves Colin, Florian Hollfelder

**Affiliations:** Department of Biochemistry, University of Cambridge, 80 Tennis Court Road, Cambridge CB2 1GA, UK

**Keywords:** bioinspiration, cell compartmentalization, synthetic biology, protein engineering, antibodies, microfluidics, emulsion droplets

## Abstract

The idea of compartmentalization of genotype and phenotype in cells is key for enabling Darwinian evolution. This contribution describes bioinspired systems that use *in vitro* compartments—water-in-oil droplets and gel-shell beads—for the directed evolution of functional proteins. Technologies based on these principles promise to provide easier access to protein-based therapeutics, reagents for processes involving enzyme catalysis, parts for synthetic biology and materials with biological components.

## 1. Introduction

The cell membrane separates molecules belonging to a cell from those that are part of the environment. A key role of this compartmentalization is to link the genotype (‘the genetic constitution of an individual’) with its corresponding phenotype (‘a set of observable characteristics of an individual’). This linkage is important for Darwinian evolution, as selection is exerted at the level of the phenotype, but survival and propagation of a selected trait are dependent on the relevant gene being carried forward to subsequent rounds of evolution. Both have to be linked to ensure that a selective advantage conferred by a gene leads to the emergence of improved species.

**Figure 1.**
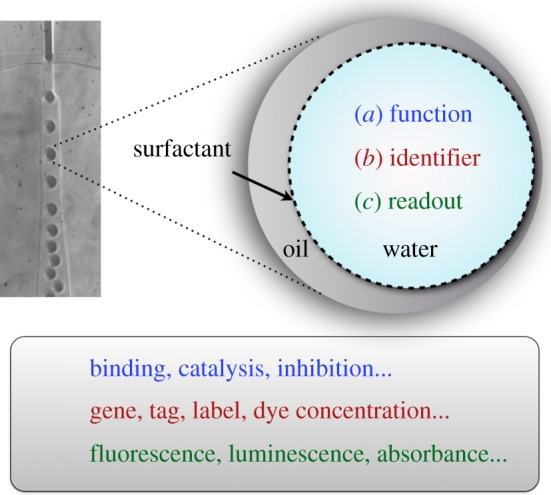
An *in vitro* compartment (a droplet [1,2] or a bead [3]) combines (*a*) the function of a molecule (e.g. the catalytic or binding activity of a protein or nucleic acid). (*b*) The information on its identity (e.g. its sequence encoded by DNA) and (*c*) a readout to assess the molecule's ability to carry out its function *via* a miniaturized assay (e.g. based on product fluorescence). Droplet diameters vary between 1 and 200 µm (corresponding to volumes between 0.5 fl and 4 nl).

It can thus be argued that compartmentalization is the organizing principle that enables Darwinian evolution—and that a cell-like compartment is the most basic ‘evolutionary unit’. This contribution describes experimental approaches towards artificial evolution that employ mimics of cellular compartments (figure 1): water-in-oil emulsions, droplets or gel beads that keep together an identifier (i.e. a gene), the functional molecule encoded by the gene (i.e. a protein) and a readout (e.g. an optical signal that distinguishes ‘winners' from ‘losers' in an evolutionary selection round). Such compartments can be made completely *in vitro*, so that—despite being inspired by cells—they are reducing the complexity of the cell-like compartment to just one purpose: linking genotype and phenotype and allowing an assay for the one function of interest. This type of biological reductionism differs from that originally proposed by Crick (‘to explain all biology in terms of physics and chemistry’) [4]: instead of a molecular understanding of the *end products* of evolution as the basis for future rational design of equally perfect or even improved constructs, the features of the engine of evolution are to be controlled, understood and ultimately mimicked: constituting a *system* that provides a route towards functional molecules.

The ability to *evolve* functional proteins is assuming an increasingly central role, because rational design of protein binders or catalysts often does not provide efficient solutions, notwithstanding the enormous progress in protein design over the last two decades. For example, antibodies used in therapy are routinely generated by ‘directed evolution’ (i.e. combinatorial selections from large libraries of candidates) and not by design despite a wealth of molecular insight into protein structure. Although we know so much, for example, about the regularity of the antibody structure and its target-binding region from comprehensive databases of primary sequences and structures, antibody binders are made by combinatorial methods (rather than by design *in silico*). Likewise, computational designs of catalytic proteins [5,6] often require further improvement by directed evolution [7,8] and the contribution of directed evolution can exceed the contribution of design [9]. Of course both approaches—design and selection—are complementary, and each directed evolution experiment will provide rational explanations and contribute in turn to the body of design rules for biological molecules. However, Crick's optimism about the supremacy of molecular design has to be mixed with a dose of realism about its limits and a realistic strategy for the creation of functional biomolecules will have to involve library methods and directed evolution.

Important criteria for a good evolution system are (i) simple to set up, (ii) allowing high throughput (more than 10^6^ experiments within a reasonable experimental timescale, i.e. hours or days) and, as far as possible, (iii) that evolution should be preferably conducted *in vitro*, because carrying out this process under non-natural conditions can overcome the following constraints:

— the requirement of having to comply with a working biological system (e.g. compatibility with a host organism)—proteins that are toxic to the host cannot be evolved;
— the narrow dynamic range of *in vivo* selections and the limit that only proteins directly relevant for survival of the host are amenable to *in vivo* evolution (e.g. in auxotrophic selections); and
— *in vivo* selections cannot be carried out under non-natural conditions, for example, involving the use of non-natural amino acids, operating at extremes of pH or temperature, or under other desired non-physiological conditions.

To free directed evolution from these constraints and drive it by arbitrarily chosen selection criteria (instead of host cell survival), attention has therefore turned to *in vitro* compartments for directed evolution that replace the cell compartment with a man-made entity that is equally suited to combine genotype and phenotype.

Joshua Lederberg anticipated the potential of such compartments in classical experiments designed to probe the clonal selection theory [10,11]: by isolating single lymph-node cells in emulsion droplet compartments, the secreted antibody was kept together with the cell producing it, thus providing genotype–phenotype linkage by compartmentalization and permitting assays to test the characteristics of each secreted protein. These groundbreaking studies provided evidence for the ‘one cell-one antibody’ rule [12]. Already at the time, Lederberg suggested that such compartments would ‘find routine applications in any laboratory’, which now, half a century later, is starting to become reality.

The potential of emulsion compartments for molecular evolution was first explored by Tawfik & Griffiths [13]. To obtain ‘monoclonal’ compartments (in which one gene and the corresponding protein encoded by it are unambiguously linked), a gene library is diluted so that each droplet contains no more than one member. Encapsulation of particles and molecules into droplets follows a Poisson distribution. In order to obtain mainly monoclonal compartments, most of the droplets are left empty. For example, a suspension containing on average 0.3 entities (DNA molecules or cells) per droplet results in 74%, 22% and 3% of the droplets containing none, one or two entities, respectively. The compartmentalization makes very large numbers of experiments possible in highly parallelized fashion, and also reduces the cost per assay dramatically (by approx. 10^6^-fold [14]), as the assay volume is reduced to the femto- to picolitre scale through use of microdroplets.

Such water-in-oil emulsion compartments can be made in a number of ways:

— by dispersing an aqueous solution in an oil phase, which produces approximately 10^9^ polydisperse droplets (diameter 1–4 µm) in one experiment—which is simply accomplished with an emulsifier or stirrer—taking only a few minutes [15–18], or
— in a microfluidic droplet generator by break-off from an aqueous stream, in which approximately 10^7^ monodisperse compartments with identical size (typically 10–200 µm, adjustable as a function of the device design and flow rates) are produced per hour [15,19,20].

## 2. Protein display systems generated in compartments

**Figure 2.**
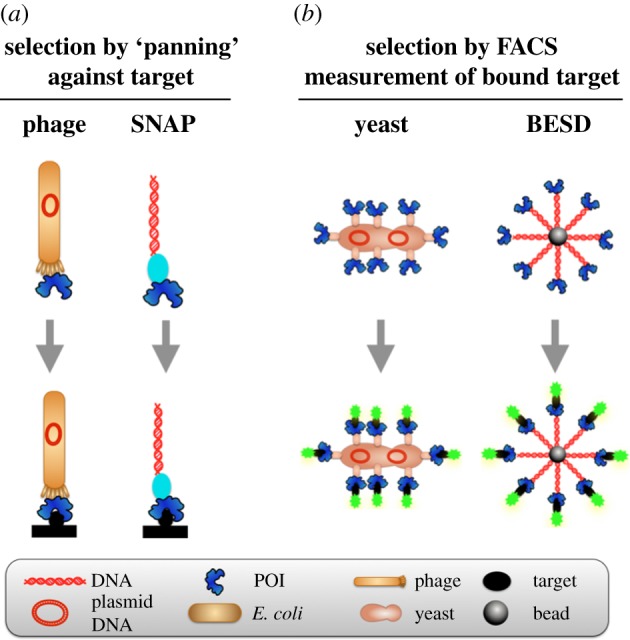
Comparison of natural and artificial display systems distinguished by the method of selection [23]. (*a*) A functional molecule (i.e. a protein of interest, POI) is connected to a gene encoding it. Selection from a library is based on binding to an immobilized target molecule: if binding molecules can be pulled out from a library based on their affinity by such ‘panning’, the attachment of the coding gene means that the selected clones can be sequenced. In this process, quantitative and direct control of ligand-binding parameters are not possible. Further, labour-intensive measurements are often necessary to assess the strength and specificity of affinity-selected binders. The natural phage display system is contrasted to *in vitro* SNAP display. (*b*) Yeast display provides multiple copies of the POI, as does its *in vitro* equivalent BeSD. Flow cytometry (FACS) measures the *number* of fluorescently labelled target molecules bound to the display construct and thus *screens* every mutant in the library, allowing a quantitative threshold to be set as the basis for a considered choice during selection.

High-affinity protein binders with defined specificity have become indispensable reagents in basic research, large-scale proteomic studies, and also in therapy, where they represent the fastest growing segment of the pharmaceutical market. The need for protein binders is addressed by display technologies [21,22]. For example, in phage display the protein of interest (POI) is fused to a coat protein, e.g. via the N-terminus of the minor (pIII) or major (pVIII) capsid proteins (figure 2) [24–26]. Protein expression occurs *in vivo*, but subsequent selections are carried out *in vitro*. It would be desirable to carry out the entire expression and selection process under *in vitro* conditions and generate a robust and stable display construct. The benefits of a cell-free format have been demonstrated by comparisons of affinity and diversity of binders generated in display formats that involve a host versus *in vitro* systems [27,28].

**Figure 3.**
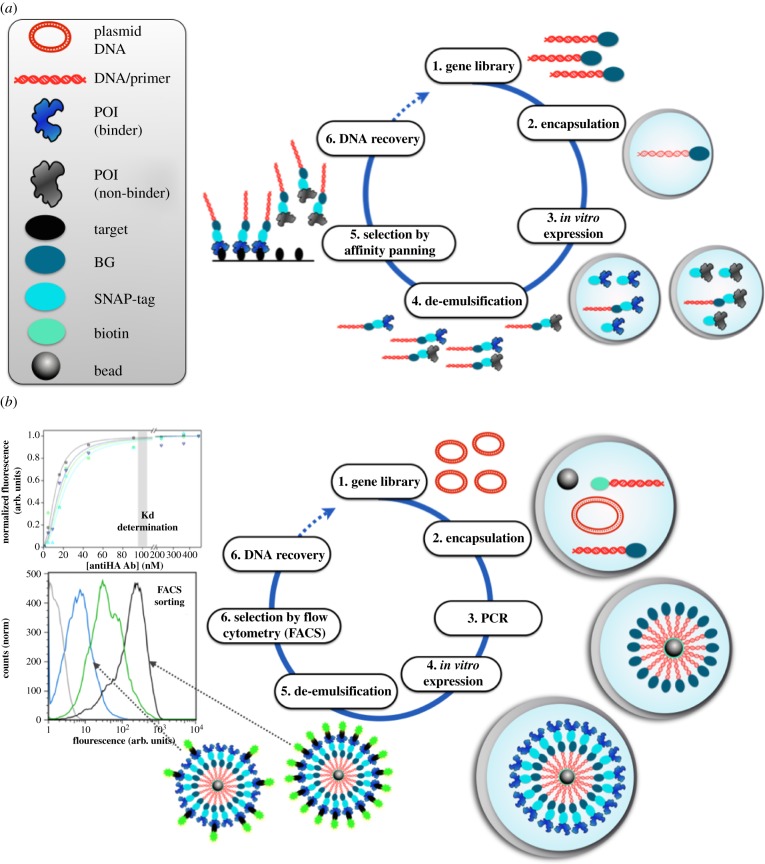
Formats for artificial covalent genotype–phenotype linkages based on droplet compartmentalization. The key initial step of both display methods is that a DNA library (coding for SNAP-tag-fused variants of the POI) is compartmentalized in water-in-oil emulsion droplets, so that each compartment contains no more than one DNA template (Poisson distribution). (*a*) In SNAP display [29–33], the POI is *in vitro* expressed from a single gene in fusion to the SNAP-tag (1). The SNAP-tag of the expressed fusion protein then reacts with its substrate, BG, that has been covalently linked to the DNA template. As a result, the SNAP-tag connects genotype and the displayed protein (responsible for the phenotype). (2) SNAP-tagged proteins are de-emulsified and challenged for binding against an antigen by affinity panning. (3) After non-binders are washed away, binders are eluted together with their encoding genes that can feed the next round of selection. (*b*) In *BeSD display* [23], the DNA is amplified by ePCR (using appropriate labelled primers), captured on the beads via a biotin–streptavidin linkage and the POI is *in vitro* expressed. After the emulsion is broken, beads are incubated with the labelled target and the affinity for the target is measured via fluorescence-activated sorting (FACS). The binding affinity of each recovered variant can be measured by subsequent FACS analysis on the bead display construct. The bead connects genotype and the megavalently displayed protein (responsible for the phenotype).

In the SNAP display [13,29,30], a display construct is assembled with the help of an *in vitro* compartment (figure 3*a*). A link between the POI and DNA is brought about by compartmentalizing a single DNA molecule in each water-in-oil emulsion microdroplet, expressing the POI *in vitro* and retaining both together by the microdroplet boundary. The corresponding protein is expressed as a fusion with a protein tag that reacts covalently with a label on its coding DNA (a benzylguanine (BG) [34] coupled to DNA) and the droplet compartment keeps gene and cognate protein together (assuring monoclonality). Inspired by the linkage of DNA and POI on a phage, the bare-bones SNAP-display is a reductionist model of the natural phage display system.

Selections are performed by ‘panning’ under *in vitro* conditions: the display construct is passed over immobilized target molecules and the binders stick (with their DNA attached—and can thus be decoded). These selections are based on off-rates (*k*_off_) and highly dependent on the conditions employed (e.g. the duration and number of washes in the panning procedure). Variants are recovered if their affinity is above a pre-set threshold, but this threshold is not necessarily precisely defined (i.e. a function of the experimental protocol and the operator's handling).

In a further variation of SNAP display, the ‘panning’ step is replaced with more quantitative, direct readouts of a binding constant (*K*_D_). When display constructs contain a larger number of proteins—e.g. approximately 10^4^ copies displayed on bacteria [35–40] or 30 000 copies on yeast [41]—selections can be based on the measurement of the number of bound target molecules (counted by quantification of an optical label for every clone): flow cytometry is employed to rank and sort binders. Variation of the concentration of a fluorescent ligand incubated with the display construct and measurement of the extent to which it sticks, determines selection pressure akin to *K*_d_ titrations. This ranking gives access to populations of weaker and stronger binders depending on the chosen fluorescence threshold in flow cytometry (figures 2*b* and 3*b*).

Inspired by yeast and bacterial display, a megavalent variation of SNAP display (dubbed BeSD, *bead surface display*) provides an *in vitro* equivalent to the multivalent natural display systems (figure 2*b*) [23]. Again, single genes are compartmentalized in emulsion droplets—but now amplification is performed in the droplet compartment and up to a million copies of DNA *and* protein are assembled on a bead in a multi-step procedure. The compartment is responsible for keeping the cognate gene and POI together and the resulting construct reminding us of yeast display, but bears more protein copies and is completely generated *in vitro*. Libraries of such constructs can now be analysed by flow cytometry and binders identified at a throughput of approximately 10^7^ per hour.

Both SNAP methods avoid shortcomings of *in vivo* display systems, e.g. low transformation efficiency, toxicity of the displayed protein to the host or lack of display construct stability.

## 3. Selections for enzyme catalysts in compartments

**Figure 4.**
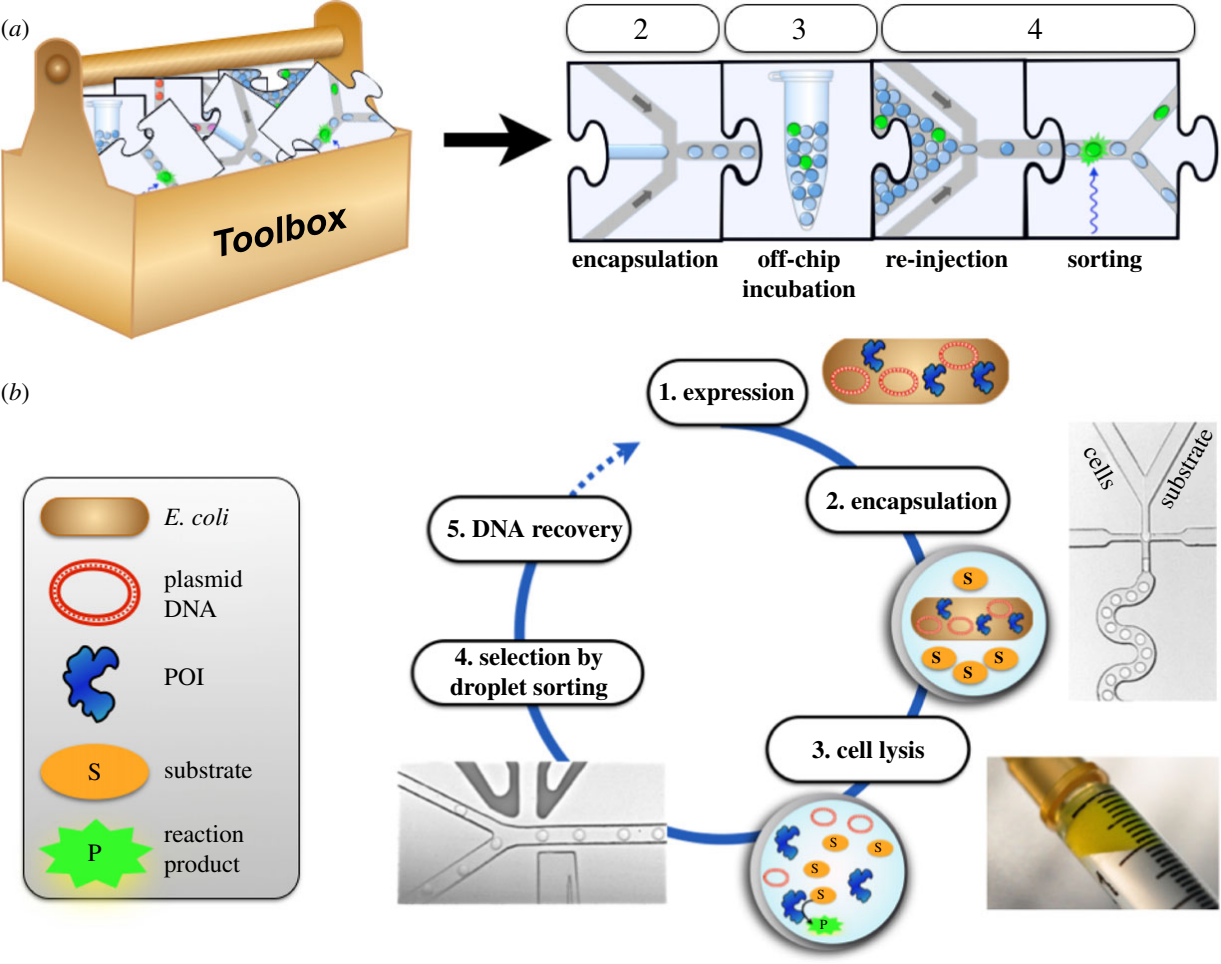
A workflow for directed evolution of a hydrolase by lysate screening in droplets [42]. (*a*) Unit operations from the ‘toolbox’ (figure 5) are assembled to miniaturize the steps necessary for single-cell assays of library members for directed evolution. (*b*) Workflow: (1) the protein of interest (POI), in this case an enzyme, is expressed in *E. coli*; (2) single cells are compartmentalized, together with substrate and cell lysis agents; (3) droplets are incubated to generate fluorescent product and (4) re-injected into a sorting device, where hits are detected by laser-induced fluorescence and steered into the upper channel by a variable electric field; (5) plasmid DNA from selected droplets is electroporated into *E. coli*. Repetition of such cycles increases the stringency of selection and enriches hits gradually to identify improved enzyme variants.

**Figure 5.**
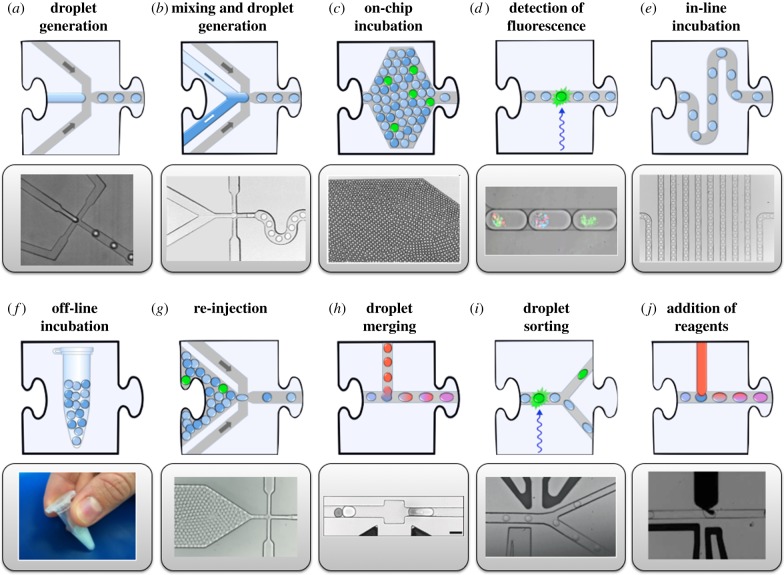
Unit operations for generating and handling *in vitro* compartments [43]. In addition to droplet formation (*a*), the microfluidic format allows a number of other unit operations. Droplets can be divided [44], fused [45–50], incubated [48,51], analysed [52–55], sorted [14,56,57] and broken up. An attractive feature of this approach is its modularity, where individual elements of a workflow correspond to experimental steps that are represented as jigsaw pieces. Each piece of the jigsaw represents a unit operation and their integration translates a macroscopic workflow to the miniaturized scale within a microfluidic device.

Instead of providing a template for the genotype–phenotype linkage that is later used for selection, the droplet compartment can also be maintained until selection, which makes it eminently suitable for selections of enzyme catalysts. Figure 4 shows how a substrate is co-compartmentalized with the protein catalyst in a droplet, multiple turnovers occur: now selections can be carried out based on product detection. To make product detection as precise as possible, microdroplets are prepared in monodisperse form in microfluidic devices (made, for example, conveniently by soft lithography from polydimethylsiloxane [15,58,59]) and interfaced with analytical systems. Figure 5 shows building blocks of integrated microfluidic devices that have recently been built. Many steps that are normally carried out in manual laboratory routines by pipetting are now automated in ‘lab-on-a-chip’ devices that process the bioinspired cell-like droplets on-chip on an assembly line at ultra-high throughput. In addition to droplet formation, the microfluidic format allows a number of other unit operations that are summarized in figure 4. Droplets are formed at rates well above 1 kHz [52,60] and can then be divided [44], fused [45–50], incubated [48,51], analysed [52–55], sorted [14,56,57] and broken up. An attractive feature of the microfluidic droplet platform is its modularity, where individual elements of a workflow correspond to experimental steps that are represented as jigsaw pieces [43]. Each piece of the jigsaw represents a unit operation and their integration translates a macroscopic workflow to the miniaturized scale within a microfluidic device. Integration of these steps with control over timing can potentially create a versatile system for directed evolution in which complex selection schemes can be realized.

Much recent work has been devoted to meeting the challenge of integration of the physical droplet processing steps with standard biological operations that may later be part of an integrated workflow for directed evolution. First, compartmentalization of cells is possible: single bacteria or yeast cells can be cultivated in droplets and recovered alive [61]. Second, *in vitro* protein expression from a single template (with up to 30 000 protein molecules expressed per DNA molecule in a droplet) has been demonstrated [51]. Kinetic parameters for several enzymes were also determined in microfluidic droplets, providing the facility to evaluate individual mutants kinetically [53,54].

**Figure 6.**
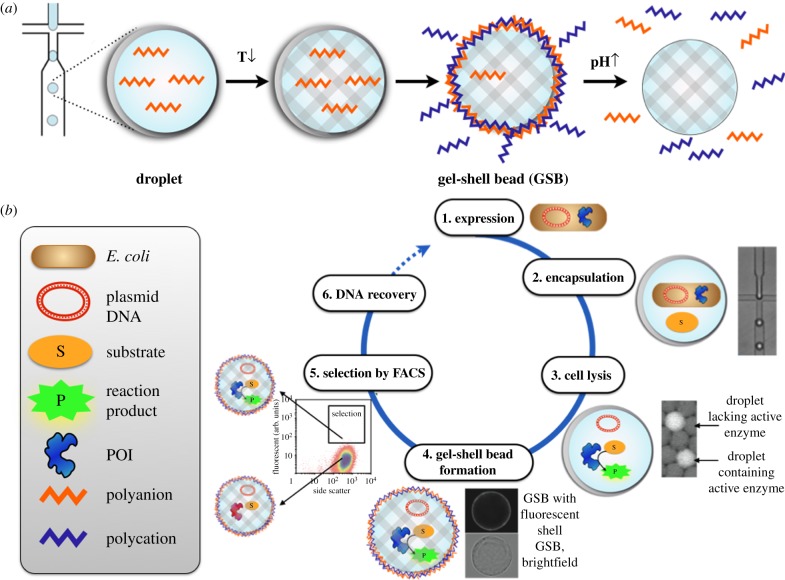
GSBs for directed enzyme evolution [3]. (*a*) Assembly of GSBs: monodisperse water-in-oil emulsion droplets are produced with a microfluidic emulsion generator. The aqueous solution from which droplets are derived contains agarose and the polyanion alginate (red zig-zag line) that gelates upon cooling (from 37°C to 4°C), so that a solid bead is formed within the droplet template. The droplet boundary is removed by breaking the emulsion in the presence of the polycation poly(allylamine hydrochloride) (PAH; blue zig-zag line). Upon spontaneous encounter of the anionic alginate and the cationic PAH at the surface of the agarose template, a polyelectrolyte complex forms that maintains compartmentalization and substitutes the oil/water interface of the former emulsion droplet with a semipermeable surface layer, functionally resembling a semipermeable cell membrane. (*b*) Workflow of directed evolution in GSBs: (1) the protein-of-interest (POI), in this case an enzyme, is expressed in *E. coli* cells prior to droplet formation. (2) Single cells are then encapsulated into monodisperse water-in-oil emulsion droplets produced by a microfluidic emulsion generator (with Poisson distribution governing the occupancy). (3) Within the droplet, cells are lysed to allow encounter with reagents and substrate: now the enzymatic reaction can take place during an incubation period. (4) The gel-droplet emulsion is broken, or de-emulsified, in the presence of PAH to form the semipermeable polyelectrolyte shell layer, (5) GSBs harbouring active enzyme are distinguished from those with inactive enzyme variants by their fluorescence (and can be selected by flow cytometry, FACS). (6) The GSBs are broken up for genotype recovery.

An entire workflow to miniaturize rounds of directed evolution is shown in figure 6: in a single-cell lysate protocol [42], single cells (each cell representing one library member) were compartmentalized with lysis reagents and substrate, so that after cell rupture compartmentalized enzymatic reactions catalysed by the protein produced by a single cell can be monitored, and subsequently sorted. Catalysts can be incubated in a delay line (with several point measurements) [42] or—for slow reactions—after offline storage for several days [62]. This procedure was exemplified by the successful evolution of a promiscuous hydrolase [42] in two rounds of genetic diversification and selection, which led to improved expression and activity by an order of magnitude each. The genotype–phenotype linkage provided by the droplet boundary was maintained until de-emulsification after selection.

## 4. Compartments turned to gel-shell beads: materials with evolvable components

Natural cells can be considered as incredible examples of functional materials, because their architecture equips them with functions of everyday survival, such as converting foodstuffs to energy, sensing or movement. In addition, they are vehicles for Darwinian evolution, providing for long-term development of an organism (or its components) by selection. By contrast, materials or devices are typically designed as such, but no mechanism for adaptation is built in. While future generations of cells will invariably evolve (e.g. in response to an environmental challenge), man-made materials will be limited to the original design: improvements are possible, but the designer has to intervene to specifically improve its properties.

In an attempt to turn droplet compartments into composite materials, we devised gel-shell beads (GSBs) that resemble minimalist versions of a natural cell [3]: a shell surrounds its interior, where functional molecules and their code (DNA) are lodged. To this end, microfluidic devices were used to produce large numbers of cell-sized droplets, which contain agarose that forms a stable structure (similar to the cytoskeleton): upon lowering the temperature, additional ingredients—agarose and alginate—solidify creating agarose microspheres (Ø ∼ 25 µm) in droplets and ‘immortalize’ the monoclonal nature of the original droplet. Addition of a functional polyelectrolyte shell with selective permeability (like the semipermeable cell membrane) completes the synthesis of biomimetic compartments. The shell—created by layer-by-layer technology [63–65]—is capable of selective retention (with permeability only for molecules less than 2 kDa) that co-compartmentalizes genotype and phenotype, thereby keeping the coding DNA, the enzyme and its (fluorescent) reaction product together. Most importantly, the beads are robust and can be easily screened/sorted using standard flow cytometry, a feature that sets GSBs apart from any existing high-throughput screening system currently available.

As above, single bacteria were encapsulated with substrate in microdroplets and lysed to liberate the POI and successful selections of a bioremediation catalyst, a phosphotriesterase, were carried out [3].

GSBs can be seen as containers for biocatalysts for cost-effective and sustainable applications that require easy recovery and repeated use of enzymes [66]. The stable catalyst cage of a GSB can contain single proteins, but may also encapsulate multiple components, e.g. sequential enzyme cascades or tandem reactions [67–71], enzymatic pathways [72–74] or synthetic gene circuits [75], that can be evolved directly in this format.

As the GSBs maintain the elements necessary for the directed evolution of an encapsulated protein, evolvability is programmed into these constructs (GSBs). The evolved catalyst is caged in GSBs, where it remains catalytically active and able to turn over multiple substrate molecules that enter the cage from the outside. After it has done its job, the caged catalyst can be removed, stored (e.g. by freezing) and used again.

In the composite GSBs, the functional components are DNA encoded, so by evolving a caged enzyme, *evolvability* of a ‘composite material’ (= a functional enzyme in a scaffold) is demonstrated: each composite carries the functional component (the enzyme) together with information that defines the identity of its functional component (DNA). This type of evolvability is key to diving further into ‘functional diversity space’. Where combinatorial approaches exist in materials science, libraries are usually smaller than screened here (almost a million members). The combination of the ability to decode a single species, extreme miniaturization (to pl droplets) and extremely straightforward screening/sorting in a commercial flow cytometer, provides the basis for easy access to molecular diversity, increasing the chances of success and setting the scene for more ambitious searches for novel functional materials.

## 5. Conclusion

The idea of cell compartmentalization is inspiring a range of practical approaches aimed at making new, functional molecules by Darwinian evolution. The extension of evolutionary principles that are enabled by the compartmentalization in its various guises has the potential to shape our material world as much as evolution has shaped Nature, with the only difference that it is up to us to decide for which purpose bioinspired parts and devices should be used.
